# Supplementary material for: The hybrid RAVE complex plays V-ATPase-dependent and -independent pathobiological roles in Cryptococcus neoformans
Source: PLoS Pathog. 2023 Oct 9;19(10):e1011721. doi: 10.1371/journal.ppat.1011721 (PMC10586682; doi:10.1371/journal.ppat.1011721)
Supplement: S4 Table — (DOCX) [file ppat.1011721.s004.docx]

**S4 Table.** **List of strains used in this study**

| Strain | Genotype | Parent | Reference |
| --- | --- | --- | --- |
| H99 | *MAT*α |  | [1] |
| YL99 | *MAT***a** |  | [2] |
| YSB10528 | *MAT*α P*_CTR4_:SKP1::NAT* | H99 | This study |
| YSB3176 | *MAT*α P*_CTR4_:TOR1*::*NAT* | H99 | [3] |
| YSB11090 | Diploid *ade2/ADE2 ura5/URA5 skp1/SKP1*::*NAT* | AI187 |  |
| AI187 | Diploid *ade2/ADE2 ura5/URA5* | JF99, M001 | [4] |
| YSB7465 | *MAT*α *rav1*Δ::*NAT-STM #125* | H99 | This study |
| YSB7589 | *MAT*α *rav1*Δ::*NAT-STM #125* | H99 | This study |
| YSB10032 | *MAT*α *wdr1*Δ::*NAT-STM #208* | H99 | This study |
| YSB10604 | *MAT*α *wdr1*Δ::*NAT rav1Δ*::*HYG* | H99 | This study |
| YSB10754 | *MAT*α *rav1*Δ::*RAV1* | H99 | This study |
| YSB10755 | *MAT*α *rav1*Δ::*RAV1* | H99 | This study |
| YSB2916 | *MAT*α *kic1*Δ::*NAT-STM#201* | H99 | [5] |
| YSB2942 | *MAT*α *cbk1*Δ::*NAT-STM#232* | H99 | [5] |
| YSB119 | *MAT*α *aca1*Δ::*NAT-STM*#43 *ura5 ACA1-URA5* | YSB108 | [6] |
| YSB121 | *MAT***a** *aca1*Δ::*NEO ura5 ACA1-URA5* | YSB109 | [6] |
| YSB10429 | *MAT***a** *rav1*Δ::*NAT-STM #125* | YL99 | This study |
| YSB10706 | *MAT***a** *wdr1*Δ::*NAT-STM #208* | YL99 | This study |
| YSB42 | *MAT*α *cac1*Δ::*NAT-STM #159* | H99 | [6] |
| YSB64 | *MAT*α *hog1*Δ::*NAT-STM #177* | H99 | [5, 7] |
| CHM3 | *MAT*α *lac1*Δ::*NAT* | H99 | [6, 8] |
| YSB2612 | *MAT*α *STE6-GFP* | H99 | [9] |
| YSB10634 | *MAT*α *STE6-GFP rav1*Δ::*NAT-STM #125* | YSB2612 | This study |
| YSB9795 | *MAT*α *rav1*Δ::*RAV1-mCherry* | YSB7589 | This study |
| YSB4095 | *MAT*α *sit4*Δ::*NAT-STM #232* | H99 | [10] |

**References**

1. Perfect JR, Ketabchi N, Cox GM, Ingram CW, Beiser CL. Karyotyping of *Cryptococcus neoformans* as an epidemiological tool. J Clin Microbiol. 1993;31(12):3305-9.10.1128/jcm.31.12.3305-3309.1993

2. Semighini CP, Averette AF, Perfect JR, Heitman J. Deletion of *Cryptococcus neoformans* AIF ortholog promotes chromosome aneuploidy and fluconazole-resistance in a metacaspase-independent manner. PLoS Pathog. 2011;7(11):e1002364.10.1371/journal.ppat.1002364

3. So YS, Lee DG, Idnurm A, Ianiri G, Bahn YS. The TOR pathway plays pleiotropic roles in growth and stress responses of the fungal pathogen *Cryptococcus neoformans*. Genetics. 2019;212(4):1241-58.10.1534/genetics.119.302191

4. Idnurm A. A tetrad analysis of the basidiomycete fungus Cryptococcus neoformans. Genetics. 2010;185(1):153-63.10.1534/genetics.109.113027

5. Lee KT, So YS, Yang DH, Jung KW, Choi J, Lee DG, et al. Systematic functional analysis of kinases in the fungal pathogen Cryptococcus neoformans. Nat Commun. 2016;7:12766.10.1038/ncomms12766

6. Bahn YS, Hicks JK, Giles SS, Cox GM, Heitman J. Adenylyl cyclase-associated protein Aca1 regulates virulence and differentiation of *Cryptococcus neoformans* via the cyclic AMP-protein kinase A cascade. Eukaryot Cell. 2004;3(6):1476-91.10.1128/EC.3.6.1476-1491.2004

7. Bahn YS, Kojima K, Cox GM, Heitman J. Specialization of the HOG pathway and its impact on differentiation and virulence of *Cryptococcus neoformans*. Mol Biol Cell. 2005;16(5):2285-300.10.1091/mbc.e04-11-0987

8. Hicks JK, D'Souza CA, Cox GM, Heitman J. Cyclic AMP-dependent protein kinase catalytic subunits have divergent roles in virulence factor production in two varieties of the fungal pathogen *Cryptococcus neoformans*. Eukaryot Cell. 2004;3(1):14-26.10.1128/Ec.3.1.14-26.2004

9. Jung KW, So YS, Bahn YS. Unique roles of the unfolded protein response pathway in fungal development and differentiation. Sci Rep. 2016;6:33413.10.1038/srep33413

10. Jin JH, Lee KT, Hong J, Lee D, Jang EH, Kim JY, et al. Genome-wide functional analysis of phosphatases in the pathogenic fungus *Cryptococcus neoformans*. Nat Commun. 2020;11(1):4212.10.1038/s41467-020-18028-0
