# Supplementary material for: The hybrid RAVE complex plays V-ATPase-dependent and -independent pathobiological roles in Cryptococcus neoformans
Source: PLoS Pathog. 2023 Oct 9;19(10):e1011721. doi: 10.1371/journal.ppat.1011721 (PMC10586682; doi:10.1371/journal.ppat.1011721)
Supplement: S5 Table — (DOCX) [file ppat.1011721.s005.docx]

**S5 Table. Primers used in this study**

| Name | Primer description | Sequence (5’ to 3’) |
| --- | --- | --- |
| B11226 | *RAV1* 5’-flanking region primer L1 | AGAGGCTTGAACCATTCC |
| B11227 | *RAV1* 5’-flanking region primer L2 | CAGATCCACTAGTTCTAGAACTCCTCTGTAGCGAACG |
| B11228 | *RAV1* 3’-flanking region primer R1 | TCCAGAATTTCACTCTTACCAGCACTTGATTTGTTGAG |
| B11229 | *RAV1* 3’-flanking region primer R2 | ACAGATGAAAGCCTCAACC |
| B11230 | *RAV1* 5’-screening primer SO | GGTTTTGCTTCTTCTGCG |
| B12020 | *RAV1* 3’-screening primer SO2 | TTGTATACTTGCAAGTTATC |
| B11231 | *RAV1* Southern blot probe primer PO | GGCTTCAAGATGAGGAAAGAG |
| B12021 | *RAV1* Internal screening primer LP | GGAGTTGGAGCAGTTGCAGA |
| B12022 | *RAV1* Internal screening primer RP | GGAAGAGATGACTGAAACCTC |
| B17133 | *RAV1* 5’-flanking region primer L2 (YL99) | GTCATAGCTGTTTCCTGACAAGTTTCTGTTCGGGAG |
| B17134 | *RAV1* 5’-flanking region primer R1 (YL99) | CTGGCCGTCGTTTTACTCAAGAGTTTCATCACCTCC |
| B14195 | *WDR1* 5’-flanking region primer L1 | TCTCCGCTAATCACGATTCC |
| B14196 | *WDR1* 5’-flanking region primer L2 | TCACTGGCCGTCGTTTTACCAGTAGGTCGCGATCATCAA |
| B14197 | *WDR1* 3’-flanking region primer R1 | CATGGTCATAGCTGTTTCCTGGACAGCACTGGCATTTGAGA |
| B14198 | *WDR1* 3’-flanking region primer R2 | AGTTGCTTTGCAAGCGTTTT |
| B14199 | *WDR1* 5’-screening primer SO | AGGGAACGAAACCTTCCTGT |
| B15031 | *WDR1* 3’-screening primer SO2 | ACATATCCATGTGCCTGCAA |
| B14200 | *WDR1* Southern blot probe primer PO | CAGATTCACGCCTTTCGTTT |
| B15032 | *WDR1* Internal screening primer LP | GGCTGCACTCTTCCAAGAAC |
| B15033 | *WDR1* Internal screening primer RP | CCATAAGCAACTCACGCTCA |
| B17893 | *WDR1* 5’-flanking region primer L2 (YL99) | GTCATAGCTGTTTCCTGCAGTAGGTCGCGATCATCAA |
| B17894 | *WDR1* 5’-flanking region primer R1 (YL99) | CTGGCCGTCGTTTTACGACAGCACTGGCATTTGAGA |
| B17872 | *WDR1RAV1* 5’-flanking region primer L2 | CACTCGAATCCTGCATGCACAAGTTTCTGTTCGGGAG |
| B17873 | *WDR1RAV1* 5’-flanking region primer R1 | TCAGGATCTTCATGGCTCCTCAAGAGTTTCATCACCTCC |
| B17766 | *SKP1 CTR4* promoter replacement primer L1 | TCCCGTGGGAATAAATTGAG |
| B17767 | *SKP1 CTR4* promoter replacement primer L2 | CACTCGAATCCTGCATGCTGTTGCTGAAATTGACTT |
| B17768 | *SKP1 CTR4* promoter replacement primer R1 | ACAACGACTTCACCAATCATGGCCGAGAAGAAGCAG |
| B17769 | *SKP1 CTR4* promoter replacement primer R2 | TGCAACCAACATCGCTACAT |
| B17770 | *SKP1 CTR4* promoter replacement primer SO | CTAGAAGGCCCTGCTTGTTG |
| B17771 | *SKP1 CTR4* promoter replacement primer PO | GACGTGGAGATGGTCGTTTT |
| B20366 | *SKP1* heterozygous 5’-flanking region primer L1 | AGGACTAGGCAAGCATGTGG |
| B20367 | *SKP1* heterozygous 5’-flanking region primer L2 | TCACTGGCCGTCGTTTTACGGATCGTTCAGCGACAATCT |
| B20368 | *SKP1* heterozygous 3’-flanking region primer R1 | CATGGTCATAGCTGTTTCCTGCTTTTGTTGCAGCCGTTAAA |
| B20369 | *SKP1* heterozygous 3’-flanking region primer R2 | CTGCATAATCAGGCGAATCA |
| B20370 | *SKP1* 5’-screening primer SO | GGTAAGATGGTGGGATGTGG |
| B20371 | *SKP1* Southern blot probe primer PO | AAGTCCGGTGTGAAAATTCG |
| B19090 | Mating locus α primer1 | ﻿GGTCTGTTGGAGGAGAAATC |
| B19091 | Mating locus α primer2 | CCTTCATCTGGCAAAAGTGAC |
| B19092 | Mating locus **a** primer1 | ﻿TGGCGAATGAAACATCGG |
| B19093 | Mating locus **a** primer2 | TCCAAGATACAAATGCTCCC |
| B1846 | M13 forward-extended primer | GTAAAACGACGGCCAGTGAGC |
| B1847 | M13 reverse-extended primer | CAGGAAACAGCTATGACCATG |
| B79 | Diagnostic screening primer1 | TGTGGATGCTGGCGGAGGATA |
| B11436 | JOHE12579 (Diagnostic screening primer2) | TTCCCACCCTCAGCAACGCC |
| B1454 | NAT split primer 1 | AAGGTGTTCCCCGACGACGAATCG |
| B1455 | NAT split primer 2 | AACTCCGTCGCGAGCCCCATCAAC |
| B1886 | NEO split primer 1 | TGGAAGAGATGGATGTGC |
| B1887 | NEO split primer 2 | ATTGTCTGTTGTGCCCAG |
| B5751 | HYG split primer 1 | CGAAGAATCTCGTGCTTTC |
| B5752 | HYG split primer 2 | ATTGACCGATTCCTTGCG |
| B15222 | CLP1 for *RAV1* complementation | ctctagatgcatgctcgagcggccgcTGGAAACGAGGAAACGGTAG |
| B15221 | CRP1 for *RAV1* complementation | AGGAAAGCAACTCTGCCAAA |
| B15220 | CLP2 for *RAV1* complementation | TTTGGCAGAGTTGCTTTCCT |
| B15223 | CRP2 for *RAV1* non-tagging complementation | cagatatccatcacactggcggccgcAGCAACGTCCAGAGGAGAAAAG |
| B13030 | Screening primer for *RAV1* complementation | AAGGCCAACTATGCGCTTC |
| B15229 | *RAV1* sequencing primer 1 | CACTAATCCACCTTATCGCA |
| B13048 | *RAV1* sequencing primer 2 | GGGCTAAAGGGTGAATAGCC |
| B13049 | *RAV1* sequencing primer 3 | TGTGGTTTAGCTCGCCGTAT |
| B13050 | *RAV1* sequencing primer 4 | ATGGACTCACACGCTTTTCA |
| B13051 | *RAV1* sequencing primer 5 | TATCCAACTCCGTCGGAAAC |
| B13052 | *RAV1* sequencing primer 6 | GGATTTCCGTGATGGTAAGG |
| B13053 | *RAV1* sequencing primer 7 | GTTGCCTGGATTTCTCCTGA |
| B13054 | *RAV1* sequencing primer 8 | TTGCGAGTCTGTCATTTTGC |
| B13055 | *RAV1* sequencing primer 9 | TGGAAGATGGGAGGAATGAG |
| B13056 | *RAV1* sequencing primer 10 | ATTCCCGTCCAGATTTCGTA |
| B13057 | *RAV1* sequencing primer 11 | ACTCGAGGTTATTGCGAGGA |
| B15228 | *RAV1* sequencing primer 12 | GTGTCAGCTGTCTAGTTTGA |
| B15351 | CRP2 for *RAV1* RFP-tagging complementation | acagagccaccgccacctgc*ggccgc*GAAGAAGTCATCCATATTAAACTCC |
| B18013 | *RAV1* qRT-PCR primer 1 | TGGCATACCGCTCCTAAATC |
| B18014 | *RAV1* qRT-PCR primer 2 | AAACAATCCCTCGTCCCTTT |
| B679 | *ACT1* qRT-PCR primer 1 | CGCCCTTGCTCCTTCTTCTATG |
| B680 | *ACT1* qRT-PCR primer 2 | GACTCGTCGTATTCGCTCTTCG |
| B8097 | *MFα1* qRT-PCR primer 1 | CGCCTTCACTGCCATCTTC |
| B8098 | *MFα1* qRT-PCR primer 2 | ACAAAGGGTCATGCCACCGG |
| B15950 | *CAP10* qRT-PCR primer 1 | TCCAGCCTTGGCTTCACTAT |
| B15951 | *CAP10* qRT-PCR primer 2 | GGCATCGAGCATAGCCTTAG |
| B8684 | *CAP59* qRT-PCR primer 1 | GCTATTAGAGGCTACAAGCG |
| B8685 | *CAP59* qRT-PCR primer 2 | GGGTGAACAACCTATCGTG |
| B8643 | *CAP60* qRT-PCR primer 1 | ACGCTATGAACGAAGAGGC |
| B8644 | *CAP60* qRT-PCR primer 2 | GGAGTGAAAACAGAGTTGGG |
| B8645 | *CAP64* qRT-PCR primer 1 | CAAGGAAAGGGCATTCAGAG |
| B8646 | *CAP64* qRT-PCR primer 2 | TCAGAAAGCATTGCCTGG |
| B14738 | *GAT201* qRT-PCR primer 1 | ATTCGGGCGACAAGAAGAAT |
| B14739 | *GAT201* qRT-PCR primer 2 | ACAATGTTCTCGGACCCATC |
| B15946 | *YAP1* qRT-PCR primer 1 | CAGAATGCAAGCCAGAATCA |
| B15947 | *YAP1* qRT-PCR primer 2 | GCGTTCATGCTGTTGTTGTT |
| B6368 | *ADA2* qRT-PCR primer 1 | TGATGCCGAAATGGCTGTAA |
| B2187 | *ADA2* qRT-PCR primer 2 | TTCATCTGGAGGACGAGTG |
| B9420 | *BZP4* qRT-PCR primer 1 | TCTTTCCCAAGTAGCATTCCTCG |
| B9421 | *BAP4* qRT-PCR primer 2 | GCTCGTCATCCCAACTATCAAAAC |
| B15938 | *LAC1* qRT-PCR primer 1 | CACCCTTTGGAAGTTGTG |
| B15939 | *LAC1* qRT-PCR primer 2 | TGATAATTGCAGAGTACCG |
| B15940 | *USV101* qRT-PCR primer 1 | CGAATCCCAGTGGAATGCC |
| B15941 | *USV101* qRT-PCR primer 2 | TCCAGCATCGTCATTGAG |
| B15942 | *MBS1* qRT-PCR primer 1 | TATCACCAATCAAGCGGC |
| B15943 | *MBS1* qRT-PCR primer 2 | TCTCCTCTTCGTTTCCCTCG |
| B15944 | *HOB1* qRT-PCR primer 1 | CCTCGCAAGTTCCCCAGCTA |
| B15945 | *HOB1* qRT-PCR primer 2 | GTATGAGGTCTTGTCCACC |
