# Supplementary figures and images for: The hybrid RAVE complex plays V-ATPase-dependent and -independent pathobiological roles in Cryptococcus neoformans
Source: PLoS Pathog. 2023 Oct 9;19(10):e1011721. doi: 10.1371/journal.ppat.1011721 (PMC10586682; doi:10.1371/journal.ppat.1011721)

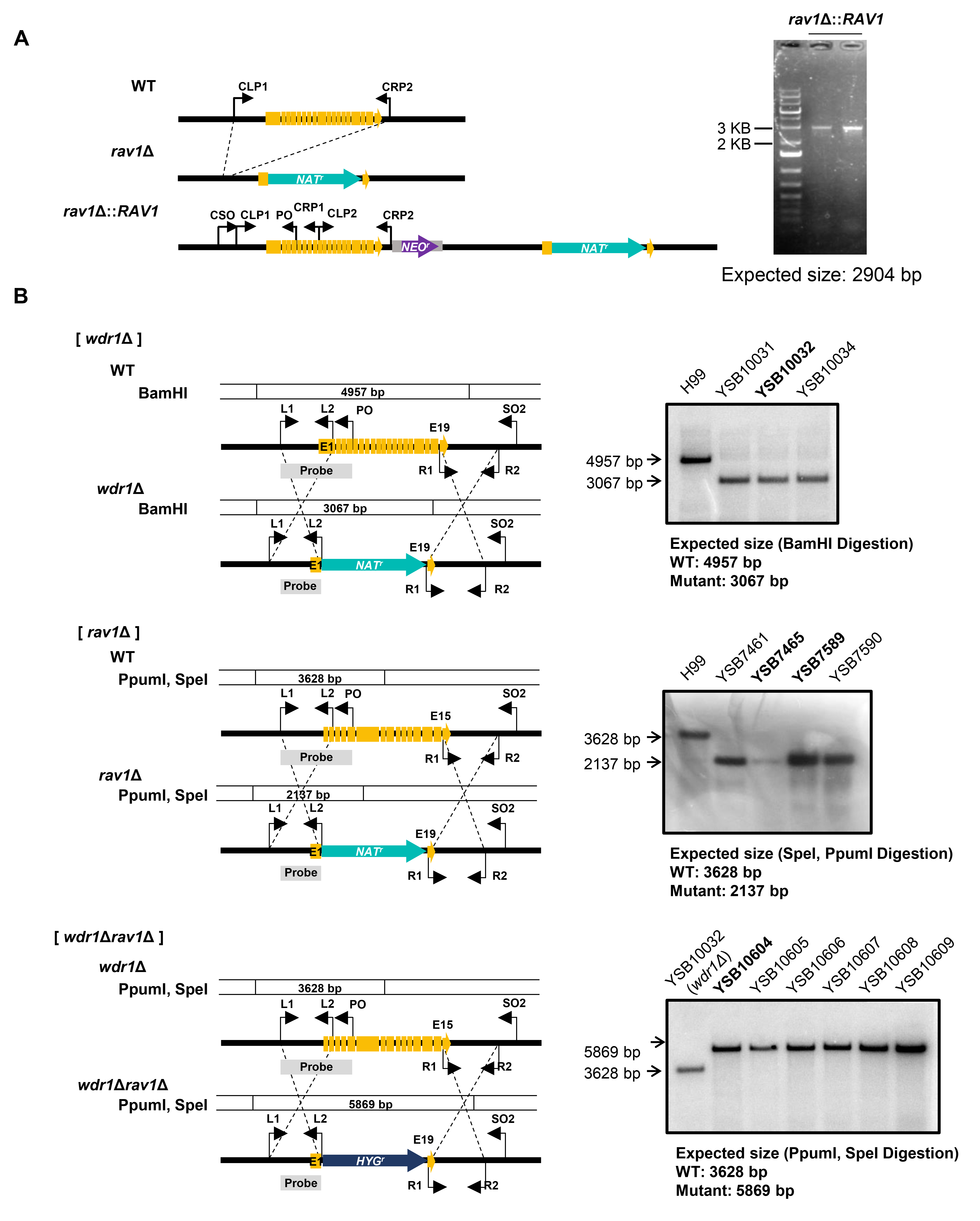

Supplement: S2 Fig — (A) Targeted integration of non-tagged RAV1 gene (rav1Δ::RAV1) was confirmed by diagnostic PCR. (B) Disruption of the WDR1, RAV1, and both genes in the MATα H99 strain and WDR1 and RAV1 in the MATa YL99 strain was confirmed by Southern blot using genomic DNAs digested with the indicated restriction enzymes. The representative strains used in this study are shown in bold. (TIF) [file ppat.1011721.s007.tif]

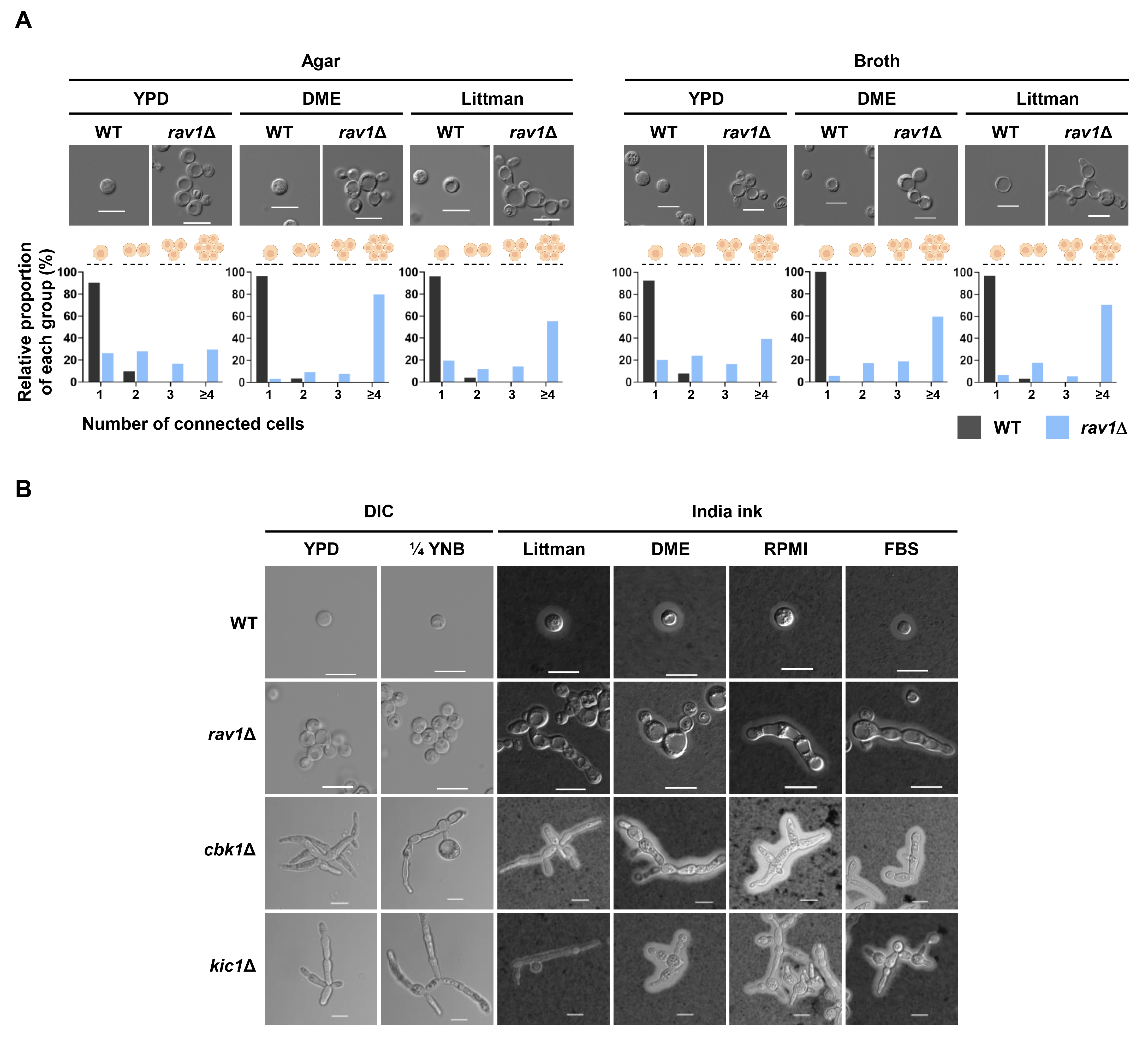

Supplement: S3 Fig — (A) Quantification of unseparated cells grown on YPD, DME, and Littman medium agar plates or liquid broth. Each C. neoformans strain was cultured at 37°C for 2 days and observed microscopically after brief sonication (>100 cell numbers). Shown are representative images of cells. (B) Morphological comparison between rav1Δ and ckb1Δ or kic1Δ in RAM pathway of C. neoformans. Each strain was cultured on YPD, YNB, Littman, DME, RPMI, and FBS agar media at 37°C for 2 days. ¼ YNB is a limiting nitrogen medium that contains 0.17% Yeast Nitrogen Base without amino acids or ammonium sulfate, 50 μM ammonium sulfate, 2% glucose, and 2% Bacto agar. Capsule was visualized by India ink staining. Scale bar indicates 10 μm. (TIF) [file ppat.1011721.s008.tif]

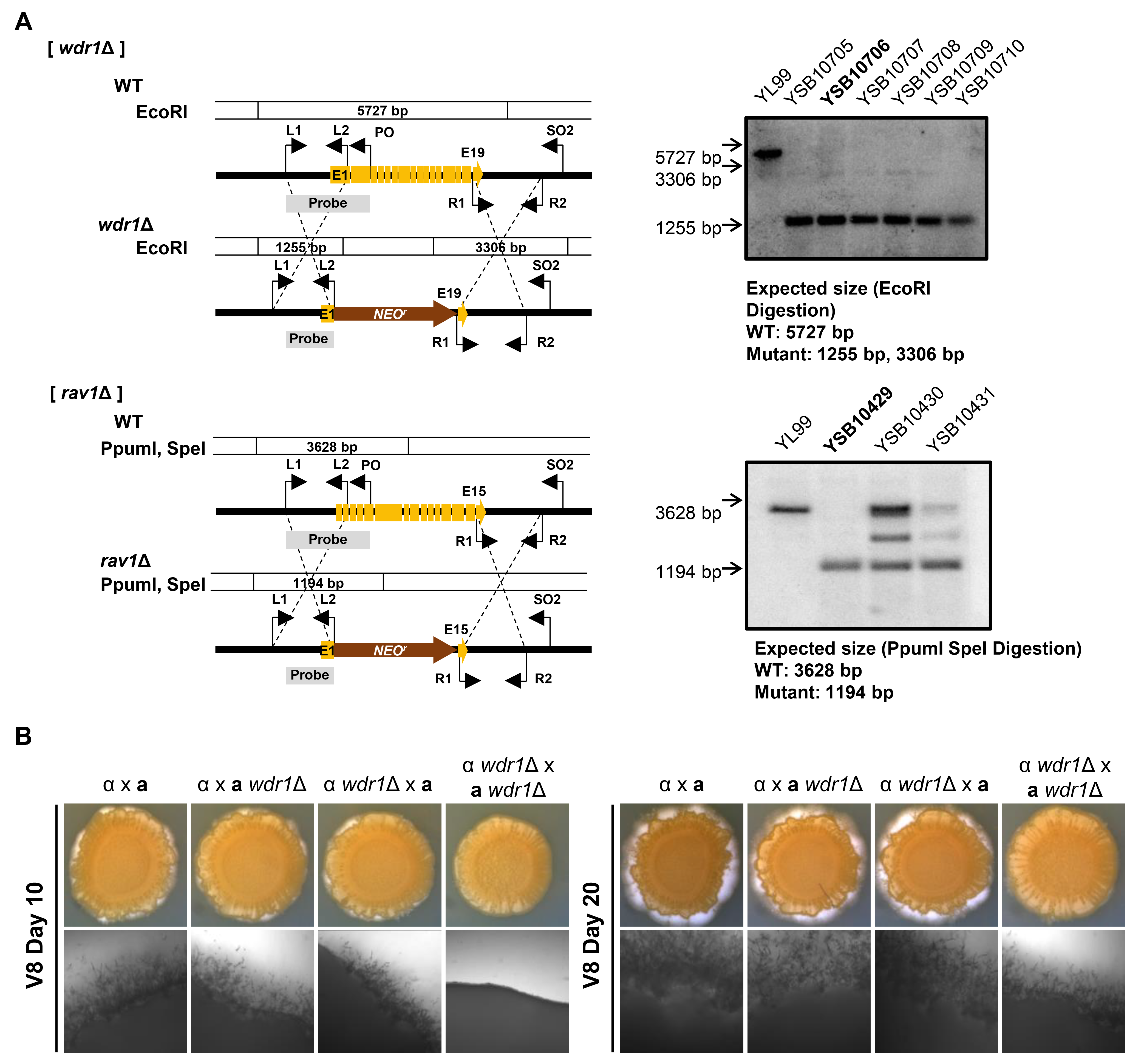

Supplement: S4 Fig — (A) Southern blot analysis confirmed the disruption of the WDR1 and RAV1 genes in the MATa YL99 strain. The representative strains used in this study are shown in bold and listed in S4 Table. (B) The indicated MATα and MATa strains were cocultured in V8 medium plates (pH 5.0) for 25 days at room temperature in the dark and photographed on the indicated days. The strains used for the mating assay are as follows: α (H99) × a (YL99), α wdr1Δ × a (YL99a), α (H99) × a wdr1Δ, and α wdr1Δ × a wdr1Δ. (TIF) [file ppat.1011721.s009.tif]

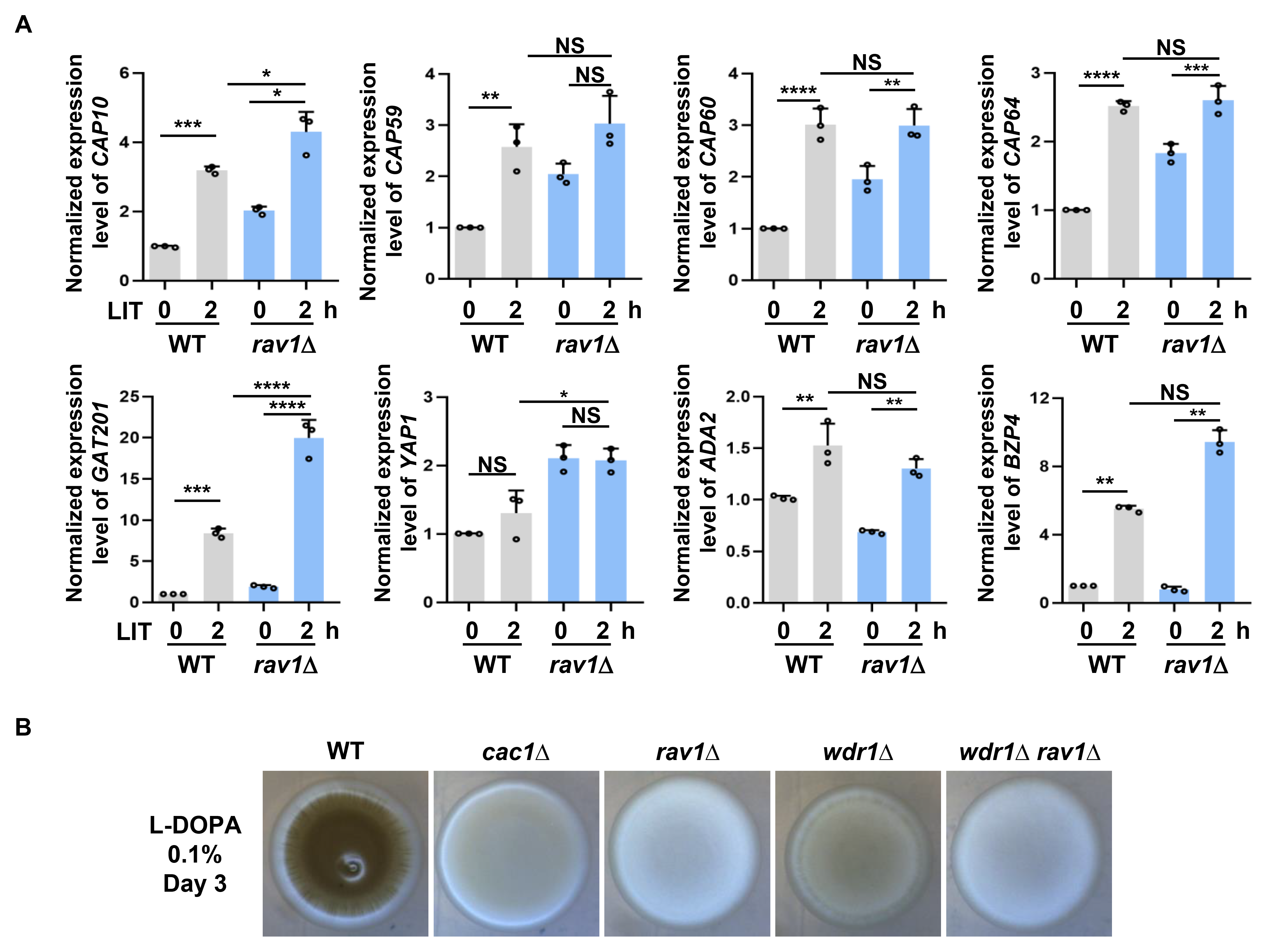

Supplement: S5 Fig — (A) The genes analyzed include CAP10, CAP59, CAP60, CAP64, GAT201, YAP1, and ADA2. Overnight cultures in YPD medium were subcultured to OD600 = 0.8 in fresh YPD medium. Each strain grown in YPD medium (time zero sample) was resuspended in Littman liquid medium, further incubated for 2 h, and extracted for total RNA. Each gene expression was normalized to ACT1 expression. The statistical significance of difference was determined using one-way ANOVA with Bonferroni’s multiple-comparison test: *, P < 0.05; **, P < 0.01; ***, P < 0.0001; NS, non-significant. Error bars indicate the standard error of the mean (SEM). (B) Melanin production assay. The wild-type (WT; H99), cac1Δ (YSB42), rav1Δ (YSB7589), wdr1Δ (YSB10032), and wdr1Δ rav1Δ (YSB10604) mutants were spotted onto L-DOPA agar medium containing 0.1% glucose, and incubated at 37°C for 3 days, and then photographed. (TIF) [file ppat.1011721.s010.tif]

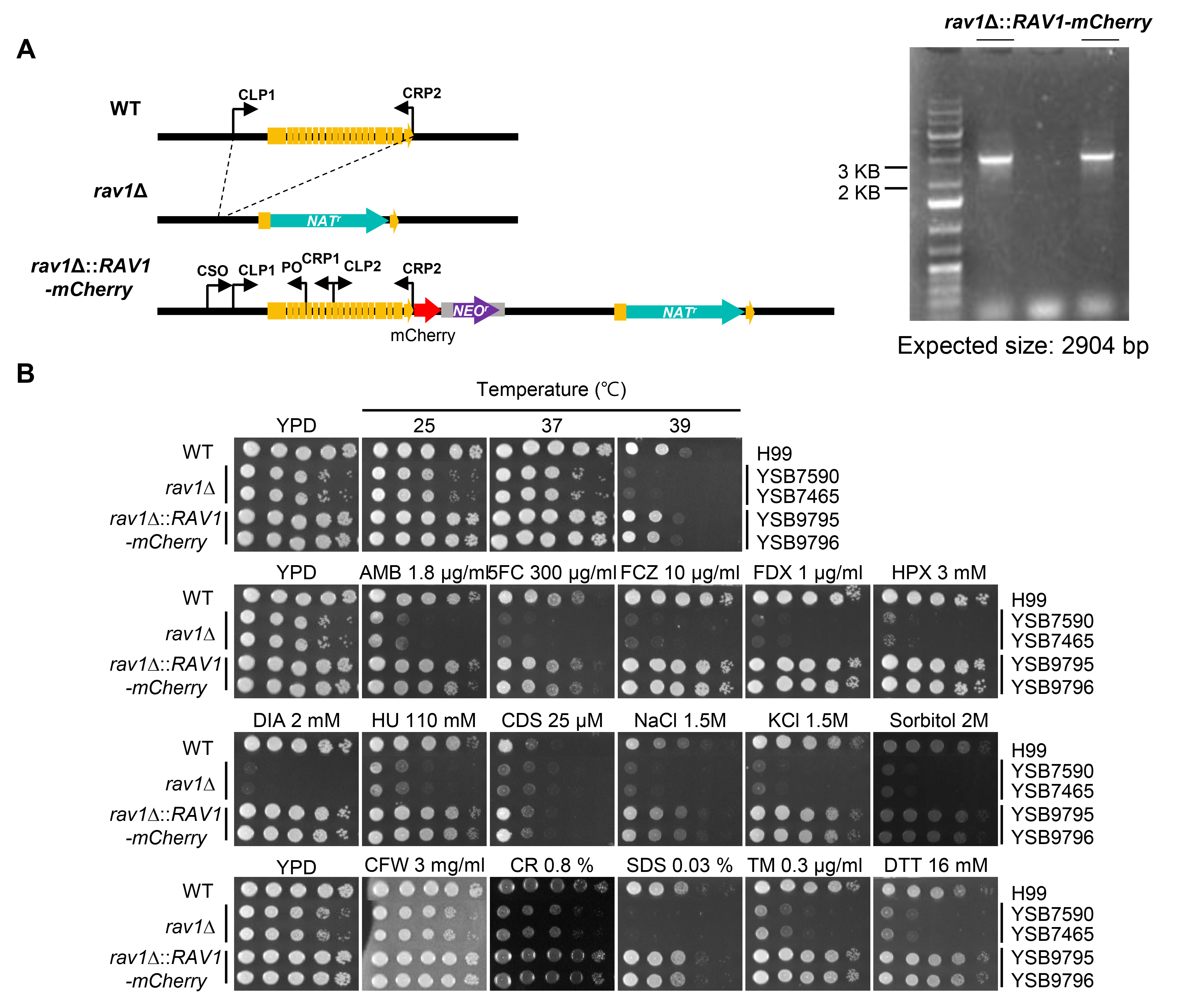

Supplement: S6 Fig — (A) Targeted integration of RAV1-mCherry tagging strains was confirmed by diagnostic PCR. (B) Phenotypic traits of the RAV1-mCherry strain were evaluated. The wild-type (WT), rav1Δ, and rav1Δ::RAV1-mCherry strains were cultured overnight in YPD broth at 30°C, serially diluted 10-fold, and spotted onto indicated stress conditions. The plates were incubated at 30°C for 3 days. (TIF) [file ppat.1011721.s011.tif]

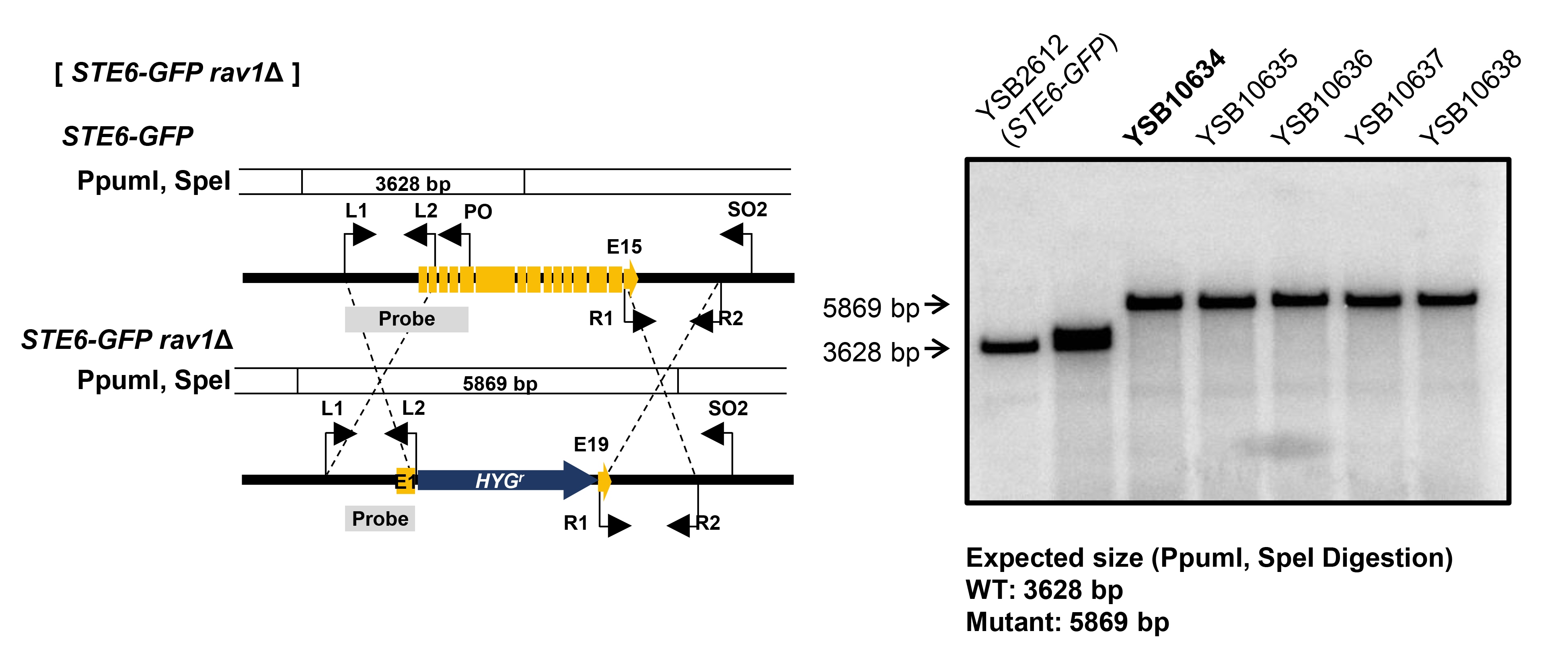

Supplement: S7 Fig — Southern blot analysis confirmed the disruption of the RAV1 in the STE6-GFP strain. Genomic DNA was digested with the indicated restriction enzymes. The representative strains used in this study are shown in bold. (TIF) [file ppat.1011721.s012.tif]

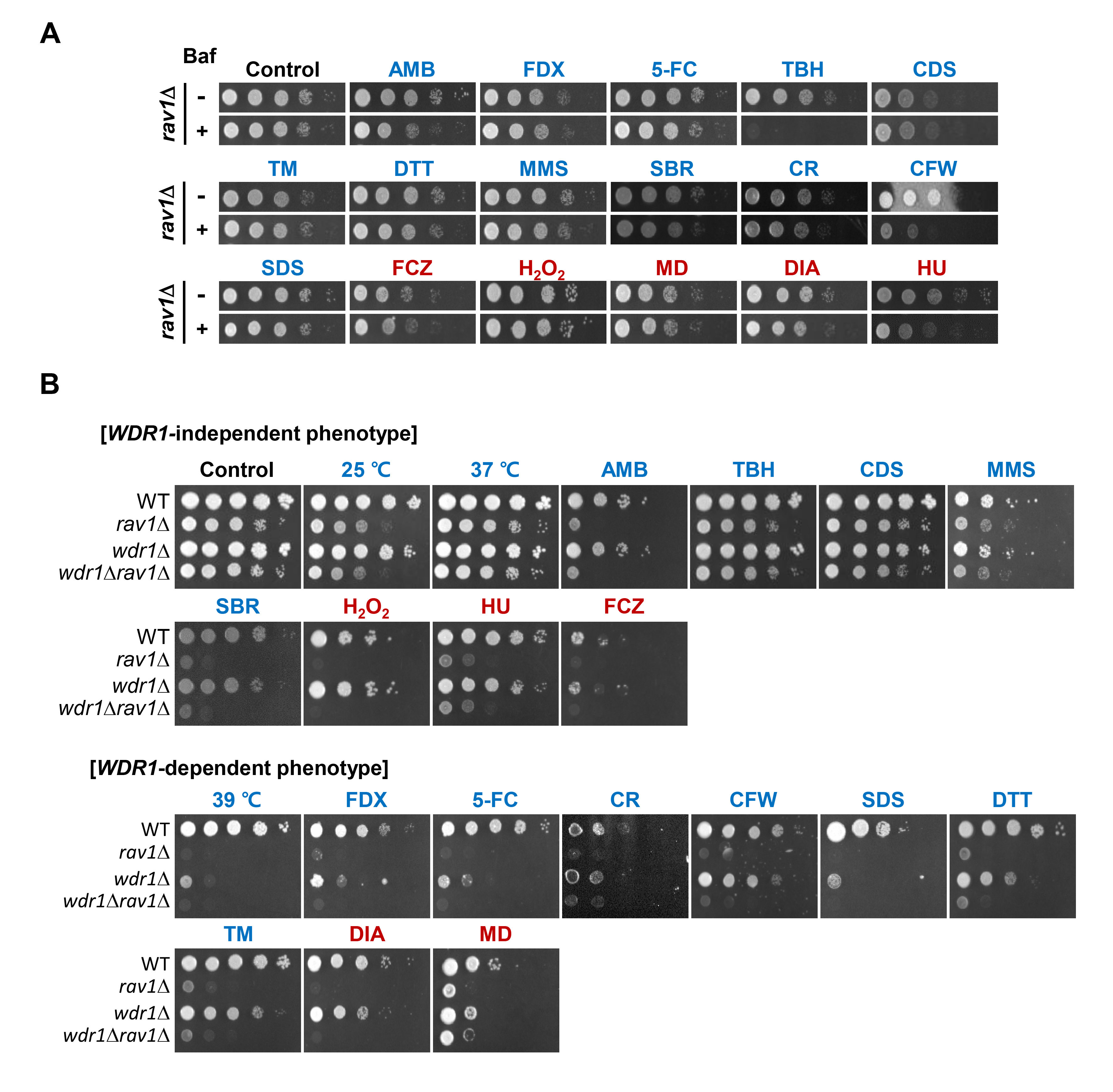

Supplement: S8 Fig — (A) Phenotypic traits of rav1Δ treated with bafilomycin A1 under following stress conditions; AMB (amphotericin B), 0.5 μg/ml; FDX (fludioxonil), 0.005 μg/ml; 5-FC (5-flucytosine), 1 μg/ml; TBH (tert-butyl hydroperoxide), 0.7 mM; CDS (cadmium sulfate), 5 μM; TM (tunicamycin), 0.3 μg/ml; DTT (dithiothreitol), 0.5 mM; MMS (methyl methanesulfonate), 0.005%; SBR (YPD + 1 M sorbitol); CR (Congo red), 0.1%; CFW (calcofluor white), 3 mg/ml; SDS (sodium dodecyl sulfate), 0.0003%; FCZ (fluconazole), 1 μg/ml; H2O2 (hydrogen peroxide), 1 mM; MD (menadione), 0.01 mM; DIA (diamide), 0.1 mM; HU (hydroxyurea), 50 mM. (B) Phenotypic traits of wdr1Δ under following stress conditions; Temperature, 25, 30, 37, and 39°C; 2.2 μg/ml AMB; 0.9 mM TBH; 25 μM CDS; 0.04% MMS; 25 μg/ml FCZ; 4 mM H2O2; 120 mM HU; SBR (YPD + 2 M sorbitol); 3 μg/ml FDX; 500 μg/ml 5-FC; 1.2% CR; 7 mg/ml CFW; 0.03% SDS; 18 mM DTT; 0.4 μg/ml TM; 2.5 mM DIA; 0.04 mM MD. Blue- and red-colored fonts indicate V-ATPase-dependent and -independent phenotypes, respectively. (TIF) [file ppat.1011721.s013.tif]

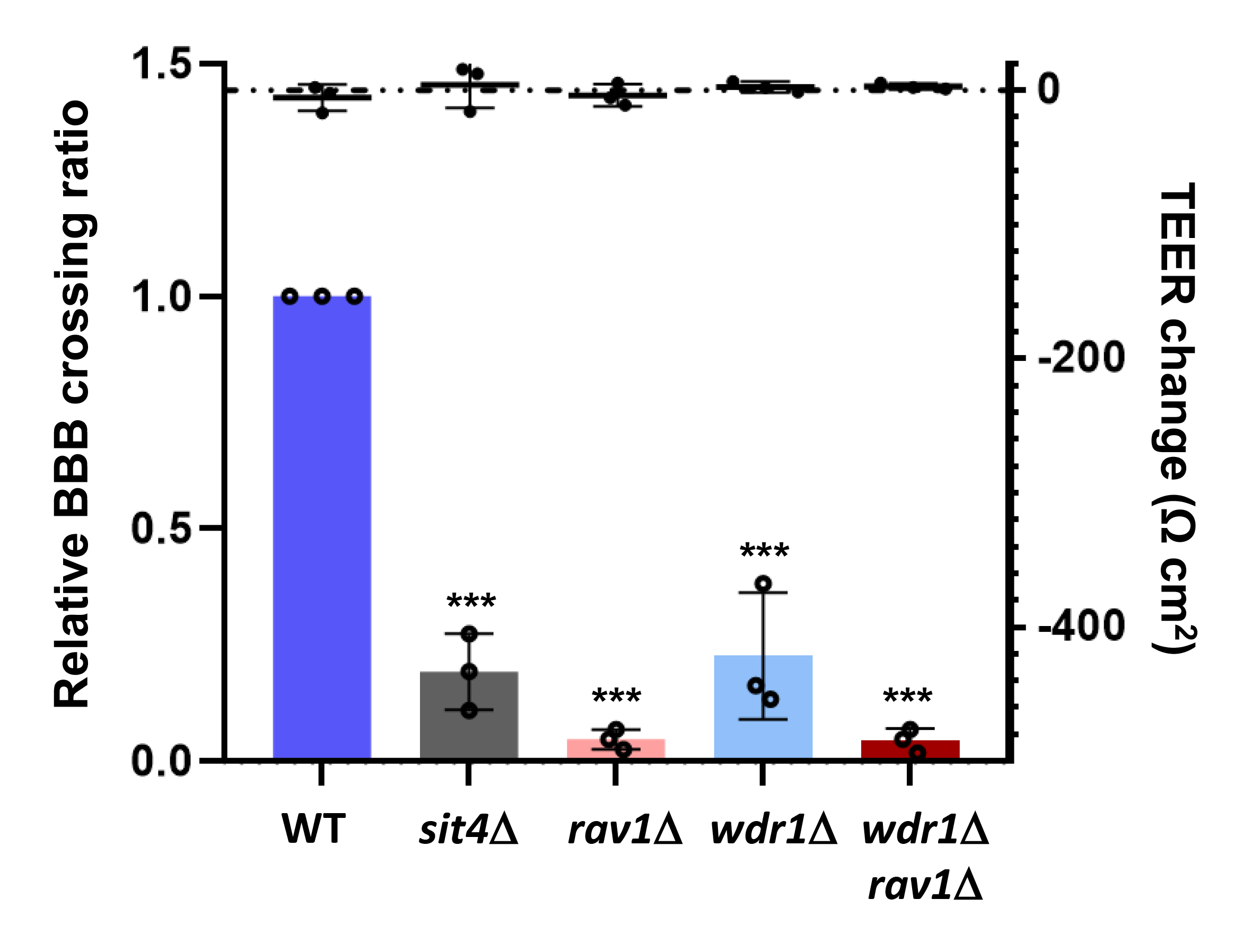

Supplement: S9 Fig — The in vitro BBB Transwell system (hCMEC/D3-coated Transwell) was used to assess the transmigration of 105 yeast cells for rav1Δ, wdr1Δ, and wdr1Δ rav1Δ mutants, incubated at 37 °C in a CO2 incubator for 24 h. The BBB crossing efficiency was calculated as outlined in the Materials and Methods section. The left Y-axis represents the relative BBB crossing ratio, which indicates the normalized BBB crossing efficiency for each tested strain relative to the wild-type (WT) strain. The right Y-axis displays the trans-endothelial electrical resistance (TEER). The sit4Δ mutant was used as a negative control. The statistical significance of the differences was determined using one-way ANOVA with Tukey’s multiple-comparison test: **, P < 0.01; ***, P < 0.0001. Error bars indicate SEM. (TIF) [file ppat.1011721.s014.tif]
