# Supplementary material for: The hybrid RAVE complex plays V-ATPase-dependent and -independent pathobiological roles in Cryptococcus neoformans
Source: PLoS Pathog. 2023 Oct 9;19(10):e1011721. doi: 10.1371/journal.ppat.1011721 (PMC10586682; doi:10.1371/journal.ppat.1011721)
Supplement: Attachment — Submitted filename: Response letter_Choi et al 2023.docx [file ppat.1011721.s016.docx]

**[Responses to reviewers’ comments]**

**REVIEWER COMMENTS**

**Part II – Major Issues: Key Experiments Required for Acceptance**

**Reviewer #1** (Remarks to the Author):

There are no major issues that need to be addressed.

**Reviewer #2** (Remarks to the Author):

1) Experimental evidence linking rav1∆ phenotypes to V-type ATPase would strengthen this study. How does bafilomycin A1 treatment impact rav1∆ phenotypes? For those that are V-type ATPase dependent, you would expect to see no difference in mutant phenotype.

**Response:** Thank you for this constructive comment. The concentration of bafilomycin A1 used in this study was 500 nM, which is known to specifically inhibit V-ATPase in previous studies (Daniel J. Klionsky et al 2008 Autophagy;849-50; Bowman E.J. et al 1988 Proc Natl Acad Sci U.S.A.;7972-7976). Therefore, we treated the *rav1*∆ strain with bafilomycin A1 at this concentration to observe its effect on phenotypes (S8A Fig). Our results showed that, under most stress conditions, *rav1*∆ exhibited similar growth to the control (YPD) even in the treatment with bafilomycin A1. However, in some stress responses, amphotericin B, *tert*-butyl hydroperoxide, calcofluor white, and hydroxyurea, we observed an increased growth inhibitory effect in *rav1*∆ upon bafilomycin A1 treatment. This observation suggests that the RAVE complex is not the exclusive regulator of V-ATPase assembly in *C. neoformans*.

In yeast, previous reports have indicated that the RAVE complex regulates the assembly of Vph1-containing V-ATPase, while the assembly of Stv1-containing V-ATPase is independent of the RAVE complex (Anne M. Smardon et al 2014 Mol Biol Cell;356-367). Additionally, Vma21 has been implicated in the initial stage of V_0_ subunit assembly (Per Malkus et al 2004 Mol Biol Cell;5075-5091), indicating the involvement of other regulators in V-ATPase assembly. Therefore, the disruption of the RAVE complex does not necessarily lead to the complete blockade of all V_1_ and V_0_ subunit assemblies. Consequently, the *rav1*∆ mutants exhibited a more severe growth defect than the untread *rav1*∆ mutant under certain conditions, potentially due to the complete inhibition of V-ATPase activity under those stress conditions. These findings suggest that V-ATPase assembly is governed by intricate regulatory mechanisms, encompassing both RAVE-dependent and independent pathways, and the specific impact of RAVE disruption varies depending on the stress context. Further investigations are warranted to fully comprehend the complexity of V-ATPase assembly regulation in response to various stress conditions. This information was added to the revised manuscript as follows:

**Lines 373-376**: The concentration of bafilomycin A1 utilized in this study was 500 nM, which has been previously reported to specifically inhibit V-ATPase at this dose [40, 41]. By treating the wild-type strain with this inhibitor, we aimed to determine if it could lead to *rav1*∆-like phenotypic changes in wild-type *C. neoformans*.

**Lines 398-406**: To further elucidate the V-ATPase-dependency of the hRAVE complex, we conducted the same experiments on the *rav1*Δ mutant (S8A Fig). In most V-ATPase-dependent phenotypes, the growth rate of the *rav1*Δ mutant treated with bafilomycin A1 showed no significant difference from the untreated control, indicating that *rav1*Δ plays a crucial role as a regulator of V-ATPase assembly. However, under certain stresses, such as amphotericin B, tert-butyl hydroperoxide, calcofluor white, and hydroxyurea, we observed further growth inhibition in the *rav1*Δ mutant upon bafilomycin A1 treatment. This suggests the possible involvement of other regulators in V-ATPase assembly under these specific stresses in *C. neoformans*.

**Lines 497-505**: Furthermore, we utilized the FungiDB (https://www.fungidb.org/) to perform BLAST analyses on Basidiomycota, and the results consistently revealed the same pattern in 34 out of the 35 Basidiomycota species (S5 Table). Nevertheless, similar to *S. cerevisiae* [24], it is conceivable that *C. neoformans* might have RAVE-independent mechanisms for regulating V-ATPase assembly. In this context, the acidification process may be slower or less efficient in the absence of the hRAVE complex, but basal-level assembly may still occur without its involvement. Further investigations are warranted to explore the existence and significance of such RAVE-independent pathways in V-ATPase assembly in *C. neoformans*.

2) There is no investigation of the role of SKP1 in the C. neoformans RAVE complex. The study relies primarily on data from the humans and S. cerevisiae to argue it is unlikely to be involved. However, additional evidence is necessary to determine whether it is truly uninvolved in C. neoformans. Also, the growth defect after SKP1 gene repression is modest – have the authors tried to knock out this gene in C. 1neoformans?

**Response:** Thank you for this constructive comment. In our LC-MS data, Skp1 was not identified as a binding partner when Rav1 was pulled down, indicating that it is not a subunit of the RAVE complex in *C. neoformans*. Furthermore, we attempted to create a knockout mutant for the *SKP1* gene multiple times but did not obtain any correct knockout strains. In addition, we confirmed that the repressed expression of *SKP1* using the *CTR4* promoter replacement system evidently reduced the growth of *C. neoformans*. As the reviewer requested to corroborate that Skp1 is an essential gene in *C. neoformans*, we generated a heterozygous mutant in AI187, a previously reported genetically engineered diploid strain [28]. Although we analyzed over 50 spores through random sporulation assay, we could not identify any *SKP1* knockout haploid spores, indicating *that* *SKP1* is indeed an essential gene in *C. neoformans*. This information was added to the revised manuscript as follows:

**Lines 164-166**: Similarly, our multiple knockout attempts of *SKP1* in the haploid *C. neoformans* H99 strain failed to yield correct knockout mutants.

**Lines 169-176**: To more definitively establish the essential role of Skp1 in the viability of *C. neoformans*, we generated heterozygous *SKP1/skp1*Δ mutants in AI187, a genetically engineered diploid strain [28] (S1E Fig). Despite examining over 50 spores through random spore analysis, we were unable to isolate any haploid spores with an *SKP1* knockout, thereby confirming the essential nature of Skp1 in *C. neoformans* (S1F Fig). Given these findings and existing reports suggesting that human Skp1 is unlikely to interact with DMXL1 and DMXL2 [23], we opted not to further investigate Skp1’s role in functionally characterizing the RAVE complex.

**Lines 631-659:** **Construction of heterozygous *SKP1*/*skp1***Δ **mutants and random spore analysis** For the generation of heterozygous *SKP1/skp1*Δ mutants, we utilized homologous recombination techniques in the genetically engineered diploid *C. neoformans* strain AI187 (*ade2/ADE2 ura5/URA5 MAT***a***/MAT*α). Gene disruption cassettes containing the nourseothricin resistance marker (*NAT*) were used. The knockout construction and validation protocol follow those established for haploid knockout strains. Targeted gene deletion was verified through Southern blot analysis (see S1E Fig in the supplemental material). For sporulation induction, the constructed *SKP1/skp1*Δ mutants were cultured overnight in liquid YPD medium at 30°C. We spotted 3 µl of 10^7^ cells/ml onto V8 mating medium (pH 5) and incubated the plates in the dark at room temperature (25°C) for over 30 days. For spore isolation, we followed the protocol previously reported by Frerichs et al. [74] with a slight modification. Following incubation on the mating medium, spores were harvested by scraping and resuspended in 75% Percoll buffer (Sigma-Aldrich, P1644). Spore suspensions were vigorously vortexed for 10 seconds until fully resuspended. Subsequently, samples were centrifuged at 3,000 rpm (1,977 x *g*) at 4°C for 30 min. The bottom of the tube was sterilized with 70% ethanol, and a needle was employed to puncture it. Five drops were collected, followed by the addition of 1.2 ml of PBS. Tubes were centrifuged again for 5 min at 3,000 rpm, and the supernatant was discarded, retaining 100 µl of buffer. An additional 1.4 ml of PBS was added for washing, and this step was repeated twice. For spore plating and verification, spores were then diluted at a 1/100 ratio, and 50 µl of this diluted spore suspension was plated onto YPD medium supplemented with 5-fluoroorotic acid (5-FOA; 100 µg/ml), adenine (20 µg/ml), and uracil (40 µg/ml). Plates were incubated at 30°C for 3 days, after which emerging colonies were streaked onto YPD plates containing chloramphenicol. After 2 days, isolated spores were spotted on various media, including YPD, YPD with nourseothricin (100 μg/ml), YNB, YNB with uracil, YNB with adenine, and YNB with both uracil and adenine. To confirm the haploid status and mating types, diagnostic PCR targeting the mating locus was performed on isolated spores. For those grown in nourseothricin-supplemented YPD, diagnostic PCR was conducted to confirm the successful internal gene deletion.

**Lines 1198-1202:** The BBB crossing efficiency was calculated as outlined in the Materials and Methods section. The left Y-axis represents the relative BBB crossing ratio, which indicates the normalized BBB crossing efficiency for each tested strain relative to the wild-type (WT) strain. The right Y-axis displays the trans-endothelial electrical resistance (TEER).

**Lines 1323-1329:** (E) BBB crossing assay. The number of yeast cells that either transcytosed through or did not transcytose through the hCMEC/D3-coated Transwell was quantified using colony-forming units (CFU) for each category. The BBB crossing efficiency was calculated as outlined in the Materials and Methods section. The left Y-axis represents the relative BBB crossing ratio, which indicates the normalized BBB crossing efficiency for each tested strain relative to the wild-type (WT) strain. The right Y-axis displays the trans-endothelial electrical resistance (TEER).

3) Expanding the analysis for Wdr1 in RAVE-dependent processes. Are the V-type ATPase-independent phenotypes dependent on Wdr1? Or does Rav1 function independent of other RAVE complex components? Are the wdr1∆ phenotypes also rescued by acidic pH?

**Response:** Thank you for this constructive comment. To thoroughly analyze *WDR1*, we repeated a series of chemical susceptibility assays for the wild-type, *rav1*∆, *wdr1*∆*,* and *rav1*∆ *wdr1*∆ strains. Since the growth difference between the wild-type and *wdr1*∆ was not evident at the previously used drug concentrations, we increased the drug dosage (FCZ 25 μg/ml; H_2_O_2_ 4 mM; HU 130 mM; MD 0.04 mM; DIA 2.5 mM) and repeated the spotting assay. Our results showed that *WDR1* is weakly involved in stress responses to menadione (MD) and diamide (DIA) among the V-ATPase-independent phenotypes, while it did not affect stress responses to fluconazole (FCZ), hydrogen peroxide (H_2_O_2_), and hydroxyurea (HU) (refer to the image attached below). In conclusion, our results indicate that under V-ATPase-independent stress responses, Rav1 and Wdr1 can act cooperatively through interactions, or Rav1 can function independently.

In the Vma^-^ phenotype, specifically regarding pH, *rav1*∆ exhibited growth recovery under acidic conditions and showed reduced growth under alkaline conditions, like the known V-ATPase subunit mutants. Therefore, we compared the growth of *wdr1*∆ under different pH conditions and found no difference in growth between acidic and neutral pH conditions, making it difficult to determine if growth recovery occurs under acidic conditions. However, under alkaline conditions, particularly in the presence of diamide (DIA), dithiothreitol (DTT), and sodium dodecyl sulfate (SDS), *wdr1*∆'s growth showed a significant decrease compared to acidic conditions (refer to the image attached below). This observation strongly suggests that Wdr1 regulates V-ATPase assembly as part of the RAVE complex.

This information was added to the revised manuscript as follows:

**Lines 407-412**: Furthermore, our findings corroborate that these V-ATPase-independent stress responses are modulated either through a Wdr1-dependent pathway, as observed with menadione or diamide treatment, or via Wdr1-independent pathway, evident with fluconazole, hydrogen peroxide, or hydroxyurea treatment (S8B Fig). Therefore, in the context of V-ATPase-independent stress responses, Rav1 and Wdr1 may collaborate or Rav1 can function independently.

**Lines 1181-1193:** **S8 Fig. Phenotypic analysis of the hRAVE complex.** (A) Phenotypic traits of *rav1*Δ treated with bafilomycin A1 under following stress conditions; AMB (amphotericin B), 0.5 µg/ml; FDX (fludioxonil), 0.005 µg/ml; 5-FC (5-flucytosine), 1 µg/ml; TBH (*tert-butyl* hydroperoxide), 0.7 mM; CDS (cadmium sulfate), 5 µM; TM (tunicamycin), 0.3 µg/ml; DTT (dithiothreitol), 0.5 mM; MMS (methyl methanesulfonate), 0.005%; SBR (YPD + 1 M sorbitol); CR (Congo red), 0.1%; CFW (calcofluor white), 3 mg/ml; SDS (sodium dodecyl sulfate), 0.0003%; FCZ (fluconazole), 1 µg/ml; H_2_O_2_ (hydrogen peroxide), 1 mM; MD (menadione), 0.01 mM; DIA (diamide), 0.1 mM; HU (hydroxyurea), 50 mM. (B) Phenotypic traits of *wdr1*Δ under following stress conditions; Temperature, 25, 30, 37, and 39°C; 2.2 µg/ml AMB; 0.9 mM TBH; 25 µM CDS; 0.04% MMS; 25 µg/ml FCZ; 4 mM H_2_O_2_; 120 mM HU; SBR (YPD + 2 M sorbitol); 3 µg/ml FDX; 500 µg/ml 5-FC; 1.2% CR; 7 mg/ml CFW; 0.03% SDS; 18 mM DTT; 0.4 µg/ml TM; 2.5 mM DIA; 0.04 mM MD. Blue- and red-colored fonts indicate V-ATPase-dependent and -independent phenotypes, respectively.

**Part III – Minor Issues: Editorial and Data Presentation Modifications**

**Reviewer #1** (Remarks to the Author):

Minor issues are reported on the marked up copy of the manuscript.

**Response:** Thank you for your constructive comments. We edited the parts you marked up in the manuscript and incorporated the changes into the manuscript. we revised as follows:

*** Major revises:**

**Reviewer #1**: Here and throughout the manuscript it would be better to say "Saccharomyces "rather than "yeast" since Cryptococcus is also a yeast.

**Original (Line 19-21):** While RAVE (regulator of H^+^-ATPase of vacuolar and endosomal membranes) and Raboconnectin-3 complexes assemble V-ATPase subunits in **yeast** and humans,

**Response** **(Line 20-22)**: “While RAVE (regulator of H^+^-ATPase of vacuolar and endosomal membranes) and Raboconnectin-3 complexes assemble V-ATPase subunits in ***Saccharomyces cerevisiae*** and humans,”

**Reviewer #1**: Change the following words to plural; 'transport', 'proton'

**Original (Line 67-70):** In humans, the V-ATPases generate a membrane potential that drives neurotransmitter uptake and transport proton across the plasma membrane in some specialized cells like osteoclasts, renal intercalated cells, and epididymal clear cells [4-10].

**Response** **(Line 68-71):** In this sentence, the context is that V-ATPase transports protons, so only the word 'proton' has been changed to the plural form. **“**In humans, the V-ATPases generate a membrane potential that drives neurotransmitter uptake and transport proton**s** across the plasma membrane in some specialized cells like osteoclasts, renal intercalated cells, and epididymal clear cells [4-10].”

**Reviewer #1**: do you mean 1235? this is consistent with Fig 1. Also normally ranges go from lowest to highest.

**Original (Line 139-142):** We identified a Rav1 ortholog in the *C. neoformans* genome database (CnRav1). Other fungi, including *C. albicans* (Ca)*, Schizosaccharomyces pombe* (Sp), and *Ustilago maydis* (Um), also appear to have a single Rav1 ortholog, of which size ranges from 1518 to 1217 amino acids (aa) (Fig 1A).

**Response** **(Line 140-143):**  “We identified a Rav1 ortholog in the *C. neoformans* genome database (CnRav1). Other fungi, including *C. albicans* (Ca)*, Schizosaccharomyces pombe* (Sp), and *Ustilago maydis* (Um), also appear to have a single Rav1 ortholog, with size ranges from **1235 to 1518** amino acids (aa) (Fig 1A).”

**Reviewer #1**: I find this sentence confusing - please rewrite

**Original (Line 363-366):** We investigated whether the hRAVE complex mediates through V-ATPase-dependent pH regulation or its unique, V-ATPase/pH-independent functions for growth, morphogenesis, sexual differentiation, and melanin production observed previously, as well as various stress responses and adaptations.

**Response** **(Line 369-372):**  “We aimed to discern whether the hRAVE complex operates through two distinct pathways: V-ATPase/pH-dependent and -independent mechanisms. Specifically, we focused on its influence on growth, morphogenesis, sexual differentiation, melanin production, and various stress responses and adaptations.”

**Reviewer #1**: Not sure this is really evident for FCZ

**Original (Line 386-387):** Notably, the increased susceptibility of *rav1*Δ to fluconazole and H_2_O_2_ also appeared to be pH-independent phenotypes (Fig 6C),

**Response:** In Fig. 6C, we observed that fluconazole susceptibility was increased in the *rav1*Δ mutant compared to the control, regardless of pH conditions. This finding is consistent with the phenotype of the *rav1*Δ mutant observed for H_2_O_2_ stress.

**Reviewer #1**: Please provide a reference for this. How many have basidiomycetes have been examined?

**Original (Line 476-477):** This pattern also occurred in the majority of Basidiomycota species.

**Response (Line 497-500): “**Furthermore, we utilized the FungiDB (https://www.fungidb.org/) to perform BLAST analyses on Basidiomycota, and the results consistently revealed the same pattern in 34 out of the 35 Basidiomycota species (S5 Table).”

**Reviewer #1**: please rephrase

**Original (Line 524-526):** Nevertheless, given that organelle acidification results from the action of V-ATPase (or V-ATPase activity), simply adjusting the extracellular pH to acidic conditions does not necessarily restore the V-ATPase function.

**Response (Line 552-554):** “However, it is important to note that while V-ATPase activity is responsible for organelle acidification, merely altering the extracellular pH to acidic conditions may not suffice to fully restore V-ATPase function.”

**Reviewer #1**: meaning?

**Original (Line 524-526):** Cytosolic pH regulation is important for fungal pathogenicity, and it is known to regulate the filamentation which is a **representative** tissue invasion mechanism of *C. albicans* [64].

**Response (Line 582-583): “**Cytosolic pH regulation is important for fungal pathogenicity, and it is known to regulate filamentation**,** which is involved in the host tissue invasion of *C. albicans* [67].”

**Reviewer #1**: cultures?

**Original (Line 647-650):** Briefly, each strain was incubated in 50 ml of YPD medium at 30°C for 16 h, washed three times with PBS, and inoculated into YNB plus 0.05% glucose with 10 mM epinephrine. The **cell** with an OD600 of 0.2 was incubated at 30°C for 4 days.

**Response (Line 701-704): “**Briefly, each strain was incubated in 50 ml of YPD medium at 30°C for 16 h. The incubated cells were washed three times with PBS, adjusted to an OD_600_ of 0.2, and inoculated into YNB plus 0.05% glucose supplemented with 10 mM epinephrine. The cultures were incubated at 30°C for 4 days.”

**Reviewer #1**: please clarify

**Original (Line 682-683):** Total RNAs were extracted by the EasyBlue (Intron) as previously described TRIzol extraction method [73].

**Response (Line 737-739):** “Total RNAs were extracted as previously described [77], except using easy-Blue Total RNA extraction kit (iNtRON) instead of TRIzol extraction solution.”

*** Minor revises:**

**Original Line**

**Line 62:** keep -> maintain

**Line 77:** proton -> protons

**Line 90:** with attenuates -> attenuates

**Line 140-142:** of which -> with

**Line 226:** It -> This

**Line 241, 248:** folds -> fold

**Line 277:** deleted *LAC1* -> with deleted *LAC1*

**Line 332-333:** In yeast, deletion of any V_0_ or V_1_ subunits of V-ATPase showed a very similar phenotype, called Vma- phenotype, showing pH and calcium-sensitive growth, metal ion sensitivity, and inability to grow on non-fermentable carbon sources [3]. -> In yeast, deletion of any V0 or V1 subunits of V-ATPase **show** a very similar phenotype, called Vma- phenotype, **with** pH and calcium-sensitive growth, metal ion sensitivity, and inability to grow on non-fermentable carbon sources [3].

**Line 334:** Among Vma- phenotypes, the pH-dependent growth -> pH-dependent growth

**Line 337:** partial Vma- phenotype -> **a** partial Vma- phenotype

**Line 366:** treating bafilomycin A1-> treatment with bafilomycin A1

**Line 367-368:** in the wild-type -> in wild-type

**Line 371, 373:** also inhibited -> inhibited, also completely-> completely

**Line 380:** the effect of bafilomycin A1 treatment was different on kinds -> the effect of bafilomycin A1 treatment varied with different kinds

**Line 464:** Therefore, it remains -> It remains

**Line 480:** functional differences from yeast -> functional differences **to** yeast

**Line 529-531:** the treatment of V-ATPase inhibitor, bafilomycin A1, increased its thermosensitivity and the stress and antifungal agent susceptibility.-> treatment **with the** V-ATPase inhibitor bafilomycin A1 increased its thermosensitivity and stress and antifungal agent susceptibility

**Line 533:** Notably, H_2_O_2_ -> H_2_O_2_

**Line 535:** to maintain its virulence [56-58]. In addition, it -> to maintain its virulence [56-58]**, and** it

**Line 559:** WD40 repeats -> WD40 repeat

**Line 564:** will -> can

**Line 669:** 5μl -> 5 μl

**Line 707:** *G. mellonella* -> *Galleria mellonella*

**Line 730:** the 5 × 10^5^ cells/mouse was adjusted, inhaled nasally -> 5 × 10^5^ cells/mouse was inhaled nasally

**Line 1251:** representative data -> representative

**Reviewer #2** (Remarks to the Author):

1) The authors argue that the C. neoformans RAVE complex is a hybrid of yeast and higher eukaryotes, but the CnRAV1 ortholog is phylogenetically more closely related to human orthologs than S. cerevisiae RAV1. This could suggest that it is not a hybrid, but simply more similar to the RAVE in higher eukaryotes. More discussion and perhaps more detailed comparisons between human DXML1/2 and ScRAV1, would be helpful.

**Response:** Thank you for your constructive comments. Upon comparing the domain structures, we observed that CnRav1 shows greater similarity in protein size and domain structure to ScRav1 rather than hsDMXL1 or hsDMXL2. On the other hand, CnWdr1 exhibits similarity to hsWDR7, leading us to conclude that the CnRAVE complex exists as a hybrid form. Additionally, we have reconstructed the phylogenetic tree using the "SHOOT" tool (https://shoot.bio/). The results show that CnRAV1 clusters with other fungal species and exhibits some genetic distance from hsDMXL1 and hsDMXL2.

We have changed the phylogenetic data for Rav1 and Wdr1 in the Results section. we revised as follows:

**Lines 151-153**: Like structural differences, CnRav1 was phylogenetically closer to other fungal Rav1 orthologs than hsDMLX1 and hsDMXL2.

**Lines 1209-1214:** The domain structure and phylogenetic tree of Rav1 and Wdr1/Rav2 orthologs in eukaryotes are illustrated in panels A and B, respectively, with abbreviations indicating the corresponding species: *U. maydis* (Um), *C. neoformans* (Cn), *C. albicans* (Ca), *S. cerevisiae* (Sc), *D. melanogaster* (Dm), *H. sapiens* (Hs), *A. thaliana* (Ath), *S. pombe* (Sp), *H. capsulatum* (Hc), *A. fumigatus* (Af), *M. globosa* (Mg), *N. crassa* (Nc), and *F. graminearum* (Fg).

2) Given the striking growth defects of the rav1∆ mutant *in vitro*, it is a surprising that this strain is virulent at all in the murine infection model. Does rav1∆ acquire mutations or adapt in some way that ultimately allow for proliferation and/or dissemination within these mice?

**Response:** Thank you for your constructive comments. According to reports by Jiu Yi et al. (Transcription factor Liv4 is required for growth and pathogenesis of *Cryptococcus neoformans*, 2020, FEMS Yeast Res), *liv4*∆ mutant, like *rav1*∆ mutant, exhibited a growth defect at 30°C, and this defect became more severe at higher temperatures. Despite the growth defect, the *liv4*∆ mutant exhibits only attenuated virulence in the infection model. So, our case is not the only one. Having said that, we also agree that it is an intriguing question whether *rav1*∆ acquires mutations or adapts in some way that could contribute to proliferation or dissemination during mouse infection. Nevertheless, answering this question may require extensive genome-sequencing analysis of numerous *rav1*∆ cells isolated from the recovered mouse tissues. We think this is beyond the current study’s scope.

3) Rav1∆ defects in the BBB transcytosis assay may be due primarily to growth defects at 37˚C, rather than a specific defect in transcytosis. Instead of transcytosis to cfu’s observed with WT C. neoformans, it would be better to normalize transcytosed Cn to non-transcytosed Cn for each strain.

**Response:** Thank you for your constructive comments. As you mentioned, we modified our data format to represent the normalized form of transcytosed Cn to non-transcytosed Cn for each strain. Again, the results demonstrate the significance of the hRAVE complex in BBB crossing, independent of the growth rate (Fig 8E, S9 Fig). we revised this part as follows:

**Lines 818-822**: The BBB crossing ratio was determined using the following equation: the number of transcytosed CFU was divided by the total CFU (both transcytosed and non-transcytosed) for each tested strain. Subsequently, the ratio obtained for each tested strain was normalized to the corresponding value for the wild-type (WT) value.

$$BBB crossing ratio={{CFU}_{transcytosed}}/{{CFU}_{total (transcytosed + non-transcytosed)}}$$

**Lines 1323-1325:** The number of yeast cells that either transcytosed through or did not transcytose through the hCMEC/D3-coated Transwell was quantified using colony-forming units (CFU) for each category.
